# Palate herpes simplex virus infection

**DOI:** 10.11604/pamj.2020.35.123.18748

**Published:** 2020-04-15

**Authors:** Giovanna Mosaico, Cinzia Casu

**Affiliations:** 1 RDH, Freelancer in Brindisi, Brindisi, Italy; 2 Private Dental Practice, Cagliari, Italy

**Keywords:** Gland, Oral herpes simplex virus, palate ulcer, palate herpes simplex virus

## Abstract

Psoriasis only affecting the genital area occurs in 2-5% of subjects with psoriasis. This condition is a frequent reason for consultation with a specialist in men’s genital mucosa, since it accounts for 24% of the reasons for consultation. The gland of circumcised subjects is usually finely squamous and non-squamous in non-circumcised subjects, based on preputial moisture. Gland involvement can be diffuse and associated with an involvement of the internal face of the prepuce. We here report the case of a 30-year old patient with a family history (grandfather) of psoriasis. The patient involved in the study had a 2-month history of squamous circumferential erythematous lesions on the glans penis without pruritus or associated urinary signs and without cutaneous manifestations. Biopsy of the mucosa of the glans penis was performed which confirmed the diagnosis of psoriasis.

## Image in medicine

A 50-year-old man came to our private practice, for palate pain and difficulty swallowing. The medical history reported gastro esophageal reflux, otherwise the patient was in good health. He reported having recently suffered from a flu syndrome treated with antibiotics (amoxicillin and clavulanic acid) cortisone and anti-inflammatory drugs. At the second day of drug therapy, the patient started to have severe oropharyngeal pain with inability to eat, reflex sialorrhea and dysphagia. From an objective examination of the oral cavity, we could see vesicular lesions along the right arch of the hard palate with partial extension to the left. A diagnosis of herpetic infection was made. The erythematous and oedematous mucosa was in the ulcerative phase. Paracetamol and systemic acyclovir have been recommended to alleviate general symptoms, fever and pain. The patient was advised to not touch the palate with his hands to avoid the expansion of the virus in other facial areas. The prescribed therapy was not performed due to the increase in esophageal gastrointestinal reflux, so after 3 weeks the lesion was still present if reduced. The differential diagnosis could be thermal burn, chemical trauma, herpes zoster lesion (VZV).

**Figure 1:**
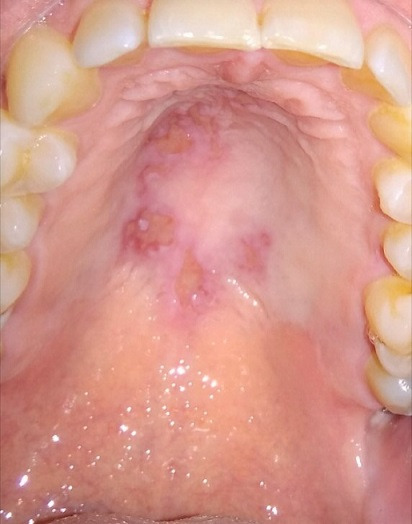
Particular manifestation of herpes simplex infection, beyond the midline

