# Corrigendum: Giovanna Mosaico *et al.* Palate herpes simplex virus infection. The Pan African Medical Journal. 2020;35:123

**DOI:** 10.11604/pamj.2020.37.25.25953

**Published:** 2020-09-07

**Authors:** The Pan African Medical Journal

**Affiliations:** 1 The Pan African Medical Journal, Yaounde, Cameroon

**Keywords:** Corrigendum, oral herpes simplex virus, palate ulcer, palate herpes simplex virus

## Abstract

This corrigendum modifies the abstract of the article: Palate herpes simplex virus infection. The Pan African Medical Journal. 2020;35: 123. doi: 10.11604/pamj.2020.35.123.18748. The error affected only the abstract of the PMC version of the article. The PDF and versions of the article on the journal website were not affected.

## Corrigendum

An error during the XML generation of the article “Palate herpes simplex virus infection” [1] resulted in the wrong abstract being included in the PMC version of the article. The error did not affect the PDF version of the article or the versions of the article available on the Journal Website. This corrigendum corrects the abstract of the Pubmed Central (PMC) version of the article only. We apologize to our readership for the inconvenience caused.
